# Multifetal Pregnancy After Implementation of a Publicly Funded Fertility Program

**DOI:** 10.1001/jamanetworkopen.2024.8496

**Published:** 2024-04-25

**Authors:** Maria P. Velez, Allison Soule, Laura Gaudet, Jessica Pudwell, Paul Nguyen, Joel G. Ray

**Affiliations:** 1 Department of Obstetrics and Gynaecology, Queen’s University, Kingston, Ontario, Canada; 2 ICES, Toronto, Ontario, Canada; 3 Department of Medicine and Obstetrics and Gynaecology, Temerty Faculty of Medicine, University of Toronto, St Michael’s Hospital, Toronto, Ontario, Canada

## Abstract

**Importance:**

A publicly funded fertility program was introduced in Ontario, Canada, in 2015 to increase access to fertility treatment. For in vitro fertilization (IVF), the program mandated an elective single-embryo transfer (eSET) policy. However, ovulation induction and intrauterine insemination (OI/IUI)—2 other common forms of fertility treatment—were more difficult to regulate in this manner. Furthermore, prior epidemiologic studies only assessed fetuses at birth and did not account for potential fetal reductions that may have been performed earlier in pregnancy.

**Objective:**

To examine the association between fertility treatment and the risk of multifetal pregnancy in a publicly funded fertility program, accounting for both fetal reductions and all live births and stillbirths.

**Design, Setting, and Participants:**

This population-based, retrospective cohort study used linked administrative health databases at ICES to examine all births and fetal reductions in Ontario, Canada, from April 1, 2006, to March 31, 2021.

**Exposure:**

Mode of conception: (1) unassisted conception, (2) OI/IUI, or (3) IVF.

**Main Outcomes and Measures:**

The main outcome was multifetal pregnancy (ie, a twin or higher-order pregnancy). Modified Poisson regression generated adjusted relative risks (ARRs) and derived population attributable fractions (PAFs) for multifetal pregnancies attributable to fertility treatment. Absolute rate differences (ARDs) were used to compare the era before eSET was promoted (2006-2011) with the era after the introduction of the eSET mandate (2016-2021).

**Results:**

Of all 1 724 899 pregnancies, 1 670 825 (96.9%) were by unassisted conception (mean [SD] maternal age, 30.6 [5.2] years), 24 395 (1.4%) by OI/IUI (mean [SD] maternal age, 33.1 [4.4] years), and 29 679 (1.7%) by IVF (mean [SD] maternal age, 35.8 [4.7] years). In contrast to unassisted conception, individuals who received OI/IUI or IVF tended to be older, reside in a high-income quintile neighborhood, or have preexisting health conditions. Multifetal pregnancy rates were 1.4% (95% CI, 1.4%-1.4%) for unassisted conception, 10.5% (95% CI, 10.2%-10.9%) after OI/IUI, and 15.5% (95% CI, 15.1%-15.9%) after IVF. Compared with unassisted conception, the ARR of any multifetal pregnancy was 7.0 (95% CI, 6.7-7.3) after OI/IUI and 9.9 (95% CI, 9.6-10.3) after IVF, with corresponding PAFs of 7.1% (95% CI, 7.1%-7.2%) and 13.4% (95% CI, 13.3%-13.4%). Between the eras of 2006 to 2011 and 2016 to 2021, multifetal pregnancy rates decreased from 12.9% to 9.1% with OI/IUI (ARD, −3.8%; 95% CI, −4.2% to −3.4%) and from 29.4% to 7.1% with IVF (ARD, −22.3%; 95% CI, −23.2% to −21.6%).

**Conclusions and Relevance:**

In this cohort study of more than 1.7 million pregnancies in Ontario, Canada, a publicly funded IVF program mandating an eSET policy was associated with a reduction in multifetal pregnancy rates. Nevertheless, ongoing strategies are needed to decrease multifetal pregnancy, especially in those undergoing OI/IUI.

## Key Points

**Question:**

What is the association between a publicly funded fertility program and multifetal pregnancy?

**Findings:**

In this population-based cohort study of 1 724 899 pregnancies in Ontario, Canada, the multifetal pregnancy rate was 1.4% in unassisted conception, 10.5% after ovulation induction or intrauterine insemination (OI/IUI), and 15.5% after in vitro fertilization (IVF). A comparison of the era before elective single embryo transfer (eSET) was promoted (ie, 2006-2011) with the era after the introduction of an eSET mandate (ie, 2016-2021) revealed that the multifetal pregnancy rate decreased from 12.9% to 9.1% after OI/IUI and from 29.4% to 7.1% after IVF.

**Meaning:**

These findings suggest that publicly funded IVF programs mandating eSET are associated with a decrease in multifetal pregnancy rates; however, additional strategies are needed to decrease multifetal pregnancy, especially after OI/IUI.

## Teaser

This cohort study examines the association between fertility treatment and multifetal pregnancy rates in a publicly funded fertility program in Ontario, Canada, from 2006 to 2021.

## Introduction

Infertility—the inability to conceive after 1 year of unprotected intercourse—affects 1 in 6 couples globally.^1^ Advances in fertility treatments have benefited many of those who otherwise would not be able to conceive but have also led to disproportionately high rates of multifetal pregnancies.^2^ Twin or higher-order multifetal pregnancy is associated with adverse outcomes, including severe maternal morbidity, preterm birth, stillbirth, cesarean delivery, and long-term neurodevelopmental conditions.^3,4,5,6,7,8^ Approximately 1% to 2% of all live births in Canada are achieved using fertility treatment.^6^ From 2005 to 2014, Ontario historically had the highest multifetal pregnancy rate in Canada of 3.6% per 100 births.^9^

When oral medications are used for ovulation induction or intrauterine insemination (OI/IUI), the rate of a multifetal pregnancy is 3% to 13%^10,11^; with injectable gonadotropins preceding IUI, that rate increases to approximately 30%.^2,10^ In the US in 2011, approximately 19% of twin pregnancies were attributable to OI or ovarian stimulation/IUI, whereas for higher-order multiple pregnancies, that proportion was 45%.^2^ Moreover, the estimated proportion of twins after in vitro fertilization (IVF) was 17%, whereas the proportion of higher-order multiple pregnancies was 32%.^2^

In the early years of IVF, it was common to transfer 2 or more embryos to increase the likelihood of a pregnancy, but this resulted in multifetal pregnancy rates as high as 30%.^12^ One way to reduce the risk of a multifetal pregnancy after IVF is by limiting the number of embryos transferred.^2^ Accordingly, elective single embryo transfer (eSET) was then promoted within clinical practice guidelines for patients with a favorable pregnancy prognosis.^13,14,15^ Given that eSET offers a safe and effective alternative to multiple embryo transfer,^16,17^ publicly funded assisted reproductive therapy (ART) programs have adopted an eSET policy, with some variations. For example, in Australia, eSET is not a mandatory practice but strongly recommended.^18^ In Sweden and Belgium, eSET is mandatory, except in exceptional circumstances.^19,20,21,22,23^ In Canada, the province of Quebec adopted a mandatory eSET policy in 2010, with some exceptions. In its first full year, there was a 60% relative decrease in the rate of multifetal pregnancies.^24^ Ontario’s publicly funded program, which offers an unlimited number of IUI cycles and 1 cycle of IVF for individuals younger than 43 years, mandated eSET starting in December 2015.^25^

In contrast to IVF, preventing a multifetal pregnancy is more challenging after OI/IUI, given the unpredictable number of follicles that may be stimulated.^26^ Hence, OI/IUI has been more difficult to regulate. Furthermore, prior epidemiologic studies of fertility treatment and multifetal pregnancy only assessed fetuses at birth, while not accounting for potential fetal reductions that may have been performed earlier in pregnancy.^27^ Accordingly, this population-based cohort study was undertaken to evaluate the association between fertility treatment and multifetal pregnancy rates in Ontario, while accounting for both fetal reductions and all births.

## Methods

This retrospective, population-based cohort study was conducted using existing linked administrative health data from Ontario, Canada (eTable 1 in Supplement 1). Data were linked using unique maternal and child identifiers and analyzed at ICES, an independent, nonprofit research institute whose legal status under Ontario’s health information privacy law allows it to collect and analyze health care and demographic data, without consent, for health system evaluation and improvement. The study followed the Strengthening the Reporting of Observational Studies in Epidemiology (STROBE) reporting guideline and was reviewed for ethical compliance by the Queen’s University Health Sciences & Affiliated Teaching Hospitals Research Ethics Board.

### Data Sources and Study Cohort Creation

This study comprised all live births and stillbirths in Ontario occurring at 20 weeks’ gestation or later, as well as fetal reductions, among pregnant women aged 18 to 50 years between April 1, 2006, and March 31, 2021. The study was restricted to individuals with valid Ontario Health Insurance Program (OHIP) coverage within 2 years before the estimated date of conception, which is most Ontarian residents. Data on race and ethnicity were not available in the study data sets.

Data were analyzed through ICES, an independent, nonprofit research institute funded by an annual grant from the Ontario Ministry of Health and the Ministry of Long-Term Care. As a prescribed entity under Ontario’s privacy legislation, ICES is authorized to collect and use health care data for the purposes of health system analysis, evaluation, and decision support. Secure access to these data is governed by policies and procedures that are approved by the Information and Privacy Commissioner of Ontario. Information on births and fertility treatment were accessed from the Better Outcomes Registry & Network (BORN) Ontario and Niday Legacy data sets (2006-2021). The BORN Ontario database captures 99% of maternal and newborn health records.^28^ The ICES data sets have been validated for sociodemographic characteristics, physician billing claims, and primary hospital diagnoses.^29^ Fetal reductions were obtained using the ICES-derived MOMBABY data set and OHIP billing codes, which are listed in eTable 2 in Supplement 1.

### Exposure Status and Outcomes 

The exposure of interest was mode of conception, categorized as follows: (1) unassisted conception (reference group), (2) OI/IUI, and (3) IVF. The primary outcome of interest was a multifetal pregnancy, namely, a twin (ie, 2 fetuses) or a higher-order (ie, ≥3 fetuses) pregnancy. Those who had a multifetal reduction to a singleton pregnancy were considered originally as twins, and those who had a multifetal reduction to a twin or more were considered as higher-order multiple pregnancies.^27^

### Covariates

We adjusted for potential factors that might confound the association between mode of conception and multifetal pregnancy, including maternal age at delivery, parity, income quintile, immigration status (immigrant or born in Canada),^30^ obesity, prepregnancy diabetes, and chronic hypertension. Obesity was defined as a maternal body mass index of 30 or greater (calculated as weight in kilograms divided by height in meters squared) or, when body mass index was not known, based on an OHIP billing code for obesity (*International Classification of Diseases, Ninth Revision* [*ICD-9*] code 278) within the 2-year period before the estimated date of conception.

### Statistical Analysis

The proportion of multifetal pregnancies by mode of conception was calculated overall and stratified by era (fiscal years April 2006 to March 2011, April 2011 to March 2016, and April 2016 to March 2021). Modified Poisson regression, with robust error variance, generated adjusted relative risks (ARRs) and 95% CIs, accounting for potentially more than 1 pregnancy in the same mother during the study period. Population attributable fractions (PAFs) were calculated as an estimate of the proportion of multifetal pregnancies in the total population that might be attributed to each type of fertility treatment. The PAF formula was as follows: PAF = 100 × Pd × (ARR − 1)/ARR), where Pd is the proportion of cases exposed to risk factor.^31^ Finally, absolute rate differences (ARDs) compared the era before eSET was promoted (ie, 2006-2011) to the era after the introduction of the eSET mandate (ie, 2016-2021).

Additional analysis 1 further assessed known maternal sociodemographic characteristics and preexistent health conditions and the associated risk of multifetal pregnancy. Additional analysis 2 assessed mode of conception and the risk of multifetal pregnancy reduction, as well as preterm birth.

Statistical significance was set at a 2-sided *P* < .05. All statistical analyses were performed using SAS, version 9.4 for UNIX (SAS Institute Inc).

## Results

Of all 1 724 899 pregnancies, 1 670 825 (96.9%) were by unassisted conception, 24 395 (1.4%) by OI/IUI, and 29 679 (1.7%) by IVF (eFigure in Supplement 1). The mean (SD) maternal age was 30.6 (5.2) years among women with unassisted conception, 33.1 (4.4) years in those with OI/IUI, and 35.8 (4.7) years in those with IVF. A greater proportion of individuals who received fertility treatment by OI/IUI or IVF tended to be older, reside in a high-income quintile neighborhood, or have preexisting health conditions, including obesity, diabetes, and chronic hypertension, in contrast to women who had unassisted conception (Table 1).

**Table 1.** Participant Characteristics by Mode of Pregnancy Conception From 1 724 899 Live Births and Stillbirths in Ontario, Canada, April 1, 2006, to March 31, 2021^a^

Characteristic	Unassisted conception (n = 1 670 825)	Ovulation induction or intrauterine insemination (n = 24 395)	In vitro fertilization (n = 29 679)
Maternal age, mean (SD), y	30.6 (5.2)	33.1 (4.4)	35.8 (4.7)
Nulliparous	686 009 (41.1)	15 512 (63.6)	19 710 (66.4)
Income quintile			
1 (Lowest)	353 527 (21.2)	3135 (12.8)	3216 (10.9)
2	331 006 (19.8)	4214 (17.3)	4816 (16.2)
3	348 824 (20.9)	5418 (22.2)	6302 (21.2)
4	356 301 (21.3)	6332 (26.0)	7723 (26.0)
5 (Highest)	281 167 (16.8)	5296 (21.7)	7622 (25.7)
Immigrant to Canada	403 368 (24.1)	5207 (21.3)	8297 (28.0)
Rural residence	134 729 (8.1)	1440 (5.9)	1184 (4.0)
Tobacco use	147 720 (8.8)	592 (2.4)	382 (1.3)
Alcohol consumption	3288 (0.2)	19 (0.1)	21 (0.1)
Substance use	28 744 (1.7)	101 (0.4)	92 (0.3)
Obesity	221 598 (13.3)	5592 (22.9)	4342 (14.6)
Prepregnancy diabetes	38 324 (2.3)	1014 (4.2)	963 (3.2)
Gestational diabetes	98 597 (5.9)	2764 (11.3)	3426 (11.5)
Chronic hypertension	46 337 (2.8)	1104 (4.5)	1319 (4.4)
Pregnancy era			
April 2006-March 2011	544 991 (32.6)	5244 (21.5)	5021 (16.9)
April 2011-March 2016	573 072 (34.3)	9195 (37.7)	10 437 (35.2)
April 2016-March 2021	552 762 (33.1)	9956 (40.8)	14 221 (47.9)

^a^ Data are presented as number (percentage) of participants unless otherwise indicated.

Overall, multifetal pregnancy rates were 1.4% (95% CI, 1.4%-1.4%) for births by unassisted conception, 10.5% (95% CI, 10.2%-10.9%) after OI/IUI, and 15.5% (95% CI, 15.1%-15.9%) after IVF (Table 2). Compared with unassisted conception, the ARR of any multifetal pregnancy was 7.0 (95% CI, 6.7-7.3) after OI/IUI and 9.9 (95% CI, 9.6-10.3) after IVF, with corresponding PAFs of 7.1% (95% CI, 7.1%-7.2%) and 13.4% (95% CI, 13.3%-13.4%). There was a decrease in multifetal pregnancy from 2006 to 2021, especially for IVF (Figure). Between the era of 2006 to 2011 and the era of 2016 to 2021, multifetal pregnancy rates decreased from 12.9% to 9.1% with OI/IUI (ARD, −3.8%; 95% CI, −4.2% to −3.4%) and from 29.4% to 7.1% with IVF (ARD, −22.3%; 95% CI, −23.2% to −21.6%) (Table 2).

**Table 2.** Association Between Mode of Conception and Risk of Any Multifetal Pregnancy

Era	No. with outcome/No. at risk	Rate, % (95% CI)	Relative risk (95% CI)		Population attributable fraction, % (95% CI)
			Unadjusted	Adjusted^a^	
**Overall: 2006-2021**					
Unassisted conception	23 749/1 670 825	1.4 (1.4-1.4)	1.0 [Reference]	1.0 [Reference]	NA
Ovulation induction or intrauterine insemination	2572/24 395	10.5 (10.2-10.9)	7.4 (7.1-7.7)	7.0 (6.7-7.3)	7.1 (7.1-7.2)
In vitro fertilization	4596/29 679	15.5 (15.1-15.9)	10.9 (10.5-11.2)	9.9 (9.6-10.3)	13.4 (13.3-13.4)
**April 2006-March 2011**					
Unassisted conception	8378/544 991	1.5 (1.5-1.6)	1.0 [Reference]	1.0 [Reference]	NA
Ovulation induction or intrauterine insemination	678/5244	12.9 (12.0-13.9)	8.3 (7.7-9.0)	7.3 (6.8-7.9)	5.6 (5.5-5.6)
In vitro fertilization	1477/5021	29.4 (28.2-30.7)	19.0 (18.1-19.9)	15.3 (14.4-16.1)	13.1 (13.1-13.2)
**April 2011-March 2016**					
Unassisted conception	7888/573 072	1.4 (1.4-1.4)	1.0 [Reference]	1.0 [Reference]	NA
Ovulation induction or intrauterine insemination	984/9195	10.7 (10.1-11.4)	7.8 (7.3-8.3)	7.3 (6.9-7.8)	7.7 (7.7-7.8)
In vitro fertilization	2117/10 437	20.3 (19.5-21.1)	14.7 (14.1-15.4)	13.4 (12.7-14.1)	17.8 (17.8-17.9)
**April 2016-March 2021**					
Unassisted conception	7483/552 762	1.4 (1.3-1.4)	1.0 [Reference]	1.0 [Reference]	NA
Ovulation induction or intrauterine insemination	910/9956	9.1 (8.6-9.7)	6.7 (6.3-7.2)	6.5 (6.0-6.9)	8.2 (8.1-8.3)
In vitro fertilization	1002/14 221	7.1 (6.6-7.5)	5.2 (4.9-5.5)	4.8 (4.4-5.1)	8.4 (8.3-8.6)

Abbreviation: NA, not applicable.

^a^ Adjusted for maternal age at delivery, income quintile, immigration status, obesity, parity, prepregnancy diabetes, and chronic hypertension.

**Figure.**
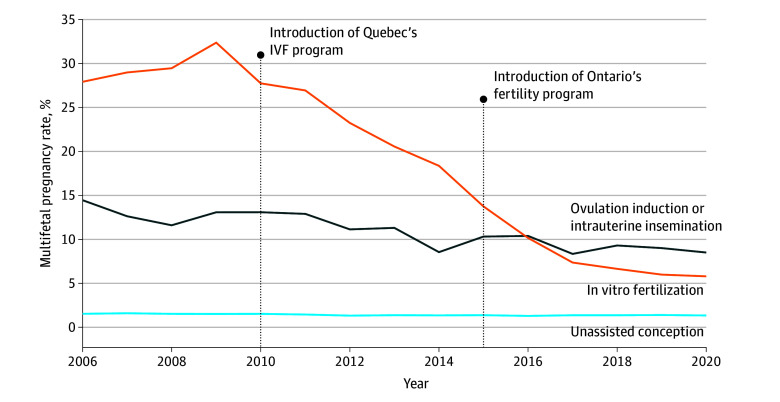
Rates of Multifetal Pregnancy by Mode of Conception and Year of the Pregnancy

Twin pregnancies occurred among 1.4% (95% CI, 1.4%-1.4%) of births by unassisted conception, 9.4% (95% CI, 9.0%-9.8%) after OI/IUI, and 14.7% (95% CI, 14.3%-15.1%) after IVF (Table 3). Compared with unassisted conception, the ARR of a twin pregnancy was 6.4 (95% CI, 6.2-6.7) after OI/IUI and 9.7 (95% CI, 9.4-10.0) after IVF, with corresponding PAFs of 6.5% (95% CI, 6.4%-6.5%) and 13.1% (95% CI, 13.1%-13.2%). Between 2006 to 2011 and 2016 to 2021, twin pregnancy rates decreased from 11.0% to 8.3% with OI/IUI (ARD, −2.7%; 95% CI, −3.1% to −2.5%) and from 27.6% to 6.8% with IVF (ARD, −20.8%; 95% CI, −21.7% to −20.0%) (Table 3).

**Table 3.** Association Between Mode of Conception and Risk of a Twin Pregnancy

Era	No. with outcome/No. at risk	Rate, % (95% CI)	Relative risk (95% CI)		Population attributable fraction, % (95% CI)
			Unadjusted	Adjusted^a^	
**Overall (2006-2021)**					
Unassisted conception	23 171/ 1 670 825	1.4 (1.4-1.4)	1.0 [Reference]	1.0 [Reference]	NA
Ovulation induction or intrauterine insemination	2292/24 395	9.4 (9.0-9.8)	6.8 (6.5-7.0)	6.4 (6.2-6.7)	6.5 (6.4-6.5)
In vitro fertilization	4356/29 679	14.7 (14.3-15.1)	10.6 (10.2-10.9)	9.7 (9.4-10.0)	13.1 (13.1-13.2)
**April 2006-March 2011**					
Unassisted conception	8115/544 991	1.5 (1.5-1.5)	1.0 [Reference]	1.0 [Reference]	NA
Ovulation induction or intrauterine insemination	577/5244	11.0 (10.2-11.9)	7.3 (6.8-8.0)	6.5 (6.0-7.0)	4.8 (4.8-4.9)
In vitro fertilization	1387/5021	27.6 (26.4-28.9)	18.4 (17.5-19.4)	14.9 (14.1-15.8)	12.8 (12.8-12.9)
**April 2011-March 2016**					
Unassisted conception	7708/573 072	1.4 (1.3-1.4)	1.0 [Reference]	1.0 [Reference]	NA
Ovulation induction or intrauterine insemination	894/9195	9.7 (9.1-10.4)	7.2 (6.8-7.7)	6.9 (6.4-7.3)	7.2 (7.1-7.3)
In vitro fertilization	2006/10 437	19.2 (18.5-20.0)	14.3 (13.6-14.9)	13.1 (12.4-13.8)	17.5 (17.4-17.5)
**April 2016-March 2021**					
Unassisted conception	7348/552 762	1.3 (1.3-1.4)	1.0 [Reference]	1.0 [Reference]	NA
Ovulation induction or intrauterine insemination	821/9956	8.3 (7.7-8.8)	6.2 (5.8-6.6)	5.9 (5.5-6.4)	7.5 (7.4-7.6)
In vitro fertilization	963/14 221	6.8 (6.4-7.2)	5.1 (4.8-5.4)	4.7 (4.4-5.0)	8.3 (8.1-8.4)

Abbreviation: NA, not applicable.

^a^ Adjusted for maternal age at delivery, income quintile, immigration status, obesity, parity, prepregnancy diabetes, and chronic hypertension.

Higher-order multiple pregnancy occurred among 0.03% (95% CI, 0.03%-0.04%) of births by unassisted conception, 1.2% (95% CI, 1.0%-1.3%) after OI/IUI, and 0.8% (95% CI, 0.7%-0.9%) after IVF (Table 4). Compared with unassisted conception, the ARR of higher-order multiple pregnancy was 29.1 (95% CI, 24.8-34.3) after OI/IUI and 19.0 (95% CI, 15.8-22.9) after IVF, with corresponding PAFs of 24.6% (95% CI, 24.5%-24.8%) and 20.7% (95% CI, 20.5%-20.9%). Between the eras of 2006 to 2011 and 2016 to 2021, higher-order multiple pregnancy rates decreased from 1.9% to 1.0% with OI/IUI (ARD, −0.9%; 95% CI, −1.2% to −0.9%) and from 1.8% to 0.3% with IVF (ARD, −1.5%; −95% CI, −1.8% to −1.2%) (Table 4).

**Table 4.** Association Between Mode of Conception and Risk of Higher-Order Multiple Pregnancy

Era	No. with outcome/No. at risk	Rate, % (95% CI)	Relative risk (95% CI)		Population attributable fraction, % (95% CI)
			Unadjusted	Adjusted^a^	
**Overall (2006-2021)**					
Unassisted conception	578/1 670 825	0.03 (0.03-0.04)	1.0 [Reference]	1.0 [Reference]	NA
Ovulation induction or intrauterine insemination	280/24 395	1.2 (1.0-1.3)	32.8 (28.4-38.0)	29.1 (24.8-34.3)	24.6 (24.5-24.8)
In vitro fertilization	240/29 679	0.8 (0.7-0.9)	23.2 (19.9-27.0)	19.0 (15.8-22.9)	20.7 (20.5-20.9)
**April 2006-March 2011**					
Unassisted conception	263/544 991	0.05 (0.04-0.05)	1.0 [Reference]	1.0 [Reference]	NA
Ovulation induction or intrauterine insemination	101/5244	1.9 (1.6-2.3)	39.0 (30.8-49.4)	30.8 (23.7-39.9)	21.5 (21.3-21.7)
In vitro fertilization	90/5021	1.8 (1.4-2.2)	36.0 (28.1-46.0)	23.7 (17.6-32.0)	19.0 (18.7-19.2)
**April 2011-March 2016**					
Unassisted conception	180/573 072	0.03 (0.03-0.04)	1.0 [Reference]	1.0 [Reference]	NA
Ovulation induction or intrauterine insemination	90/9195	1.0 (0.8-1.2)	31.1 (24.1-40.3)	24.3 (18.3-32.2)	22.7 (22.3-22.9)
In vitro fertilization	111/10 437	1.1 (0.9-1.3)	33.9 (26.7-42.9)	23.3 (17.3-31.4)	27.9 (27.5-28.2)
**April 2016-March 2021**					
Unassisted conception	135/552 762	0.02 (0.02-0.03)	1.0 [Reference]	1.0 [Reference]	NA
Ovulation induction or intrauterine insemination	89/9956	1.0 (0.7-1.1)	36.6 (28.0-47.8)	32.3 (24.0-43.5)	32.8 (32.4-33.1)
In vitro fertilization	39/14 221	0.3 (0.2-0.4)	11.2 (7.9-16.0)	9.3 (6.2-13.8)	13.2 (12.5-13.8)

Abbreviation: NA, not applicable.

^a^ Adjusted for maternal age at delivery, income quintile, immigration status, obesity, parity, prepregnancy diabetes, and chronic hypertension.

In additional analysis 1, risk factors for multifetal pregnancy are shown in eTable 3 in Supplement 1. In additional analysis 2, mode of conception was significantly associated with a higher risk of multifetal pregnancy reduction, as well as preterm birth (eTable 4 in Supplement 1).

## Discussion

In this cohort of 1.7 million pregnancies, OI/IUI and IVF were both associated with multifetal pregnancies. Although there was a decrease in the overall rate of multifetal pregnancies between 2006 and 2021, that decrease was more substantial for IVF pregnancies than for those conceived after OI/IUI.

The initial decrease between 2011 and 2016 in the rate of multifetal pregnancies in Ontario was likely explained by the corresponding recommendation for eSET, as in the guidelines published by the Society of Obstetricians and Gynaecologists of Canada and the Canadian Fertility and Andrology Society.^32^ This recommendation was paralleled by a significant decrease in multifetal pregnancy rates in the neighboring province of Quebec after public funding for IVF was introduced in 2010.^24^ After the introduction of the Ontario Fertility Program in December 2015, there was even a further decrease in the rate of multifetal pregnancies from 2016 to 2021, which was in alignment with other publicly funded IVF programs mandating eSET.^18,19,20,21,22,23,24^

The Ontario Fertility Program funds 1 cycle of IVF. Some patients decide to do a private cycle either instead of waiting for the publicly funded cycle or after a failed publicly funded IVF cycle. The Canadian Assisted Reproductive Technology Registry reported 66 994 IVF cycles in Ontario between 2016 and 2021 in Ontario.^33^ Of these, 50% were publicly funded and 50% were private. The respective pregnancy rates were 37.8% and 41.4%, and the respective multifetal live birth rates were 0.9% and 2.1%. Certainly, future studies should address the cost-effectiveness of providing 1 vs multiple publicly funded IVF cycles,^24^ especially, because some couples in Ontario still pursue privately paid IVF cycles,^33^ which can result in a higher rate of multifetal pregnancy and an inherently higher risk of maternal and neonatal morbidity.^3,27^

Improvements and advances in ART have also contributed to the success of eSET programs. Advances in embryo culture media have led to a shift in IVF practice from cleavage-stage embryo transfer to blastocyst-stage embryo transfer, resulting in higher live birth rates.^34^ Embryo cryopreservation has also been a key to implementing eSET programs because this allows the elective freezing of all available embryos, with subsequent single frozen blastocyst transfer, resulting in higher cumulative pregnancy rates.^35^

Although the Ontario Fertility Program mandated eSET for women undergoing public IVF, no equivalent requirement was made for OI/IUI. Even so, the multifetal pregnancy rate after OI/IUI decreased from 12.9% in 2006 to 2011, to 10.7% in 2011 to 2016, and to 9.1% in 2016 to 2021. Hence, it is possible that the introduction in 2015 of public IVF funding might have contributed to this slight decrease in multifetal pregnancy after OI/IUI, because some couples might have chosen to move to IVF after a few cycles of IUI had failed or as their first-line treatment.

For OI/IUI, practice recommendations have suggested using less potent ovarian stimulation therapies and cancellation of IUI cycles when the ovarian response suggests a high risk of a multifetal pregnancy.^36,37^ Choosing the right type of OI medication is important. For women with polycystic ovary syndrome, for example, OI using oral letrozole results in higher live birth rates than with oral clomiphene citrate without increasing multifetal pregnancy.^37^ In women with polycystic ovary syndrome who do not achieve a pregnancy with letrozole, injectable low-dose gonadotropins may be used as a second-line agent in OI/IUI, with careful monitoring of follicle count using ultrasonography.^37^

For couples with unexplained infertility and who undergo IUI, ovarian stimulation with oral clomiphene citrate or letrozole is recommended.^38^ Given the risk of multiple pregnancy, the use of injectable gonadotropins is generally not recommended in this context.^37,38^ In Canada, however, injectable gonadotropins are sometimes offered, with patients made aware that this regimen is associated with a higher multifetal pregnancy rate per cycle than IUI with oral agents.^39^ Controlled ovarian stimulation and IUI, with strict cancellation criteria, result in similar pregnancy and multifetal pregnancy rates compared with eSET or modified natural IVF.^40^

### Strengths and Limitations

Strengths of this study include the use a large population-based sample from a validated provincial registry,^41^ comprising all births within a publicly funded health care system. The ability to account for fetal reductions and to study not only IVF but also OI/IUI are each novel study strengths.

This study also has some limitations. Nondifferential misclassification of the study exposure was unlikely, but some individuals in the unassisted conception group may have used an oral medication for OI or ovarian stimulation without that information being recorded in the BORN database. Such misclassification would have likely resulted in an underestimation of the risk estimates in those exposed to fertility treatment. Another limitation was lack of specific details about those who underwent a fetal reduction, such as the exact number of fetuses reduced, especially when the reduction was down to a singleton pregnancy. This issue would have led to an underestimation of the number of higher-order multifetal pregnancies. The absence of information about the type of medication used for OI/IUI (oral agents vs gonadotropins) is another limitation. In fact, although epidemiologic surveillance of ART is encouraged, most national registries specifically include IVF but not IUI. Another study limitation was the lack of information about the number of embryos transferred per IVF cycle (ie, SET, eSET, double embryo transfer, or higher). To be clear, not all IVF pregnancies in Ontario occurred from within the publicly funded program; some are through private cycles and others from centers outside Canada.

## Conclusions

This study’s findings suggest that multiple factors, including changes to ART best practices and the introduction of a publicly funded IVF program in Ontario, likely were associated with a significant decrease in the risk of a multifetal pregnancy after IVF. Currently, OI/IUI appears to be the largest contributor to the risk of a woman having a multifetal pregnancy after fertility treatment. These findings support the benefit of mandating eSET for women undergoing IVF programs and highlight the need to develop a parallel widespread policy for OI/IUI as well.

## Notes

Supplement 1. **eTable 1.** Data Sources Accessed at ICES **eTable 2.** Variables Used to Define Cohort Entry, Exclusion Criteria and Study Exposure, Outcomes, and Covariates **eTable 3.** Additional Analysis 1: Factors Associated With Multifetal Pregnancy in Ontario, Canada, From April 1, 2006, to March 31, 2021 **eTable 4.** Additional Analysis 2: Association Between Mode of Conception and Risk of Multifetal Pregnancy Reduction, as Well as Preterm Birth, in Ontario, Canada, From April 1, 2006, to March 31, 2021 **eFigure.** Study Flowchart

Supplement 2.
Data Sharing Statement
