# Supplementary material for: Multifetal Pregnancy After Implementation of a Publicly Funded Fertility Program
Source: JAMA Netw Open. 2024 Apr 25;7(4):e248496. doi: 10.1001/jamanetworkopen.2024.8496 (PMC11046352; doi:10.1001/jamanetworkopen.2024.8496)
Supplement: Supplement 1. — eTable 1. Data Sources Accessed at ICES eTable 2. Variables Used to Define Cohort Entry, Exclusion Criteria and Study Exposure, Outcomes, and Covariates eTable 3. Additional Analysis 1: Factors Associated With Multifetal Pregnancy in Ontario, Canada, From April 1, 2006, to March 31, 2021 eTable 4. Additional Analysis 2: Association Between Mode of Conception and Risk of Multifetal Pregnancy Reduction, as Well as Preterm Birth, in Ontario, Canada, From April 1, 2006, to March 31, 2021 eFigure. Study Flowchart [file jamanetwopen-e248496-s001.pdf]

Supplementary Online Content

Velez MP, Soule A, Gaudet L, Pudwell J, Nguyen P, Ray JG. Fertility treatment and multifetal pregnancy. *JAMA Netw Open*. 2024;7(4):e248496. doi:10.1001/jamanetworkopen.2024.8496

eTable 1. Data Sources Accessed at ICES
eTable 2. Variables Used to Define Cohort Entry, Exclusion Criteria and Study Exposure, Outcomes, and Covariates
eTable 3. Additional Analysis 1: Factors Associated With Multifetal Pregnancy in Ontario, Canada, From April 1, 2006 to March 31, 2021
eTable 4. Additional Analysis 2: Association Between Mode of Conception and Risk of Multifetal Pregnancy Reduction, as Well as Preterm Birth, in Ontario, Canada, From April 1, 2006, to March 31, 2021
eFigure. Study Flowchart

This supplementary material has been provided by the authors to give readers additional information about their work.

© 2024 Velez MP et al. *JAMA Network Open.*

eTable 1. Data Sources Accessed at ICES

**Dataset**	**Description**	**Variable(s)**
Better Outcomes Registry & Network (BORN) Ontario	A longitudinal administrative data source that collects information related to maternal, perinatal, and newborn health in Ontario from 2006 onwards.	Pregnancy related variables: mode of conception, number of fetuses, gestational age, pre-pregnancy characteristics (maternal BMI, smoking at first pre-natal visit, any substance use)
Canadian Institute for Health Information Discharge Abstract Database (CIHI DAD)	A dataset that captures administrative, clinical, and demographic information on hospital discharges, including deaths, sign-outs, and transfers.	Patient information, age, gestational age
Ontario Health Insurance Plan Claims Database (OHIP)	A dataset that records all claims for reimbursement by Ontario physicians for inpatient and ambulatory visits, consultations and procedures. The data also include claims from optometrists for publicly funded reimbursement and from laboratories for all diagnostic tests performed.	Previous history of infertility
Registered Persons Database (RPDB)	A dataset that provides demographic information about all individuals who have received an Ontario health card number, including their date of birth, sex, and home address.	Rurality index
Ontario Hypertension Dataset (HYPER)	A dataset of all of the people in Ontario identified as having hypertension.	History of Hypertension
Ontario Diabetes Dataset (ODD)	A dataset of all of the people in Ontario diagnosed with diabetes.	History of Diabetes
Immigration, Refugees and Citizenship Canada Permanent Resident Dataset (IRCC-PR)	A dataset that provides information on permanent and temporary residents of Canada, in addition to immigration and citizenship programs.	Immigration status

© 2024 Velez MP et al. *JAMA Network Open.*

eTable 2. Variables Used to Define Cohort Entry, Exclusion Criteria and Study Exposure, Outcomes, and Covariates

Assessment	Timing	Disease or procedure or condition	Applicable codes	Data sources
**Cohort entry criterion**	April 1st, 2006 to March 21st 2021	All livebirths and stillbirths at ≥20 weeks' gestation among women aged 18-50 years.		BORN NIDAY
	Same	Fetal reduction	ICD-10-CA code for "continuing pregnancy after selective fetal reduction of one fetus or more" (code O31.12)	MOMBABY
	Same	Fetal reduction	P053 - selective fetal reduction of one or more fetuses by bipolar or unipolar cautery of umbilical cord<br>P054 - selective fetal reduction of one or more fetuses by intracardiac potassium chloride injection	OHIP
**Exclusion Criteria**	At the time of hospitalization for delivery	Maternal age <18 or >50 at time of delivery.	NIDAY: Maternal age = B_BDATE – M_BDATE (baby's birth date – mother's birth date)<br>BORN-BIS: MATAGEATSTILLORLIVEBIRTHYEARS	BORN NIDAY
	Same	Gestational age <20 or >43 weeks at time of delivery.	NIDAY: GEST (between 20 to 43)<br>BORN: GA_AT_BIRTH_WEEKS (between 20 to 43)	BORN NIDAY
	Same	Less than 2 years of OHIP eligibility prior to estimated conception date.	Estimated conception date = baby's birthdate – gestational age in weeks*7<br>Niday fields: B_BDATE and GEST<br>BORB fields:B_BDATE and GA_AT_BIRTH_WEEKS	RPDB
	Same	No valid IKN.	-	BORN
	Same	Warning in NIDAY dataset.	-	NIDAY

© 2024 Velez MP et al. *JAMA Network Open.*

	Same	Inductions for the purposes of termination.	BORN dataset: ind_for_lbr_induct_prim_id: 1014250 (Reason for induction of labour: termination of pregnancy)	BORN
	Same	Birth outside of Ontario.	BORN dataset: Pregnancies with births outside of Ontario (missing lbm_submitted_id)	BORN
	Same	No infant record in born.	BORN dataset: Pregnancies with no infant record in born	BORN
	Same	Pregnancies with year of birth 1900	-	BORN
	Same	Pregnancy loss at < 20 weeks or stillbirth > 20 weeks	BORN dataset: AGG_OUTCOME_ID = 1021035 (pregnancy loss < 20 weeks), 1021040 (pregnancy loss < 20 weeks, termination), 1021050 (pregnancy loss < 20 weeks spontaneous miscarriage), 1021070 (stillbirth at >= 20 wks, termination)	BORN
**Exposure**	At the index pregnancy	Mode of conception	BORN Field= CONCEPTION_TYPE_ID<br>Niday Field= REPASS<br><br>**Unassisted**<br>Conception type = spontaneous or unknown (1013160, 1013180 [BORN] or 1,9,0 [Niday])<br>**Ovulation Induction or Intrauterine Insemination**<br>BORN = 1013110, 1013120, 1013150<br>NIDAY = 2, 5<br>**In Vitro Fertilization**<br>BORN = 1013130, 1013140, 3000006<br>NIDAY = 3, 4	BORN
**Main Outcomes**	At the index pregnancy	Multifetal pregnancy: twin or higher order multiples	Niday: MULTGEST<br>BORN: NUMBER_OF_FETUSES_ID<br><br>**Twin**<br>BORN = 1018759<br>NIDAY = 2<br><br>**Higher Order Multiple**<br>BORN = 1018760, 1018770, 1018780, 1018790, 1018800, 1018810<br>NIDAY = ≥3	BORN<br>NIDAY

© 2024 Velez MP et al. *JAMA Network Open.*

**Covariates**	At the index of pregnancy	Maternal age	--	BORN
	Same	Income quintile	(1) Lowest quintile<br>(2) Second quintile<br>(3) Third quintile<br>(4) Fourth quintile<br>(5) Highest quintile	Census
	Same	Rurality index	(1) Urban (RIO 0-39)<br>(2) Rural (RIO >=40)	Census
	Same	Immigrant status	(1) Non-Refugee Immigrant or Refugee Immigrant<br>(2) Canadian Born	IRCC-PR
	Same	Smoking	--	BORN
	Same	Substance Use	--	BORN
	Same	Alcohol exposure	--	BORN
	Same	Maternal BMI	--	BORN
	Same	Parity		BORN

© 2024 Velez MP et al. *JAMA Network Open.*

eTable 3. Additional Analysis 1: Factors Associated With Multifetal Pregnancy in Ontario, Canada, From April 1, 2006 to March 31, 2021

Characteristic	Singleton N (%)	Multifetal Pregnancy N (%)	Unadjusted relative risk (95% CI)	Adjusted relative risk (95% CI)[a]
**Maternal age**, y				
Under 20	33,422 (2.0)	304 (1.0)	0.4 (0.4-0.5)	0.6 (0.5-0.6)
20-29	641,221 (37.9)	8822 (28.5)	0.7 (0.7-0.7)	0.8 (0.8-0.9)
30-39	947,543 (55.9)	19,567 (63.3)	1.0 (ref.)	1.0 (ref.)
40 and over	71,796 (4.2)	2224 (7.2)	1.5 (1.4-1.6)	1.0 (1.0-1.1)
**Parity**				
Nulliparous	987,088 (58.3)	16,413 (53.1)	0.8 (0.8-0.9)	1.0 (1.0-1.0)
Parous	706,732 (41.7)	14,499 (46.9)	1.0 (ref.)	1.0 (ref.)
**Income quintile**				
1-2	688,423 (40.6)	11,491 (37.2)	0.9 (0.9-1.0)	1.0 (1.0-1.0)
3	354,164 (20.9)	6380 (20.6)	1.0 (ref.)	1.0 (ref.)
4-5	651,395 (38.5)	13,046 (42.2)	1.1 (1.1-1.1)	1.0 (1.0-1.1)
**Immigrant**				
Yes	410,083 (24.2)	6789 (22.0)	0.9 (0.9-0.9)	0.8 (0.8-0.9)
No	1,255,551 (74.1)	23,693 (76.6)	1.0 (ref.)	1.0 (ref.)
**Tobacco use**				
Yes	146,487 (8.6)	2207 (7.1)	0.8 (0.8-0.9)	1.1 (1.0-1.1)
No	1,459,932 (86.2)	26,537 (85.8)	1.0 (ref.)	1.0 (ref.)
**Alcohol consumption**				
Yes	3299 (0.2)	29 (0.1)	0.5 (0.4-0.7)	0.6 (0.4-0.9)
No	1,589,991 (93.9)	28,627 (92.6)	1.0 (ref.)	1.0 (ref.)
**Substance use**				
Yes	28,535 (1.7)	402 (1.3)	0.8 (0.7-0.9)	1.0 (0.9-1.1)
No	1,575,838 (93.0)	28,438 (92.0)	1.0 (ref.)	1.0 (ref.)
**Obesity**				
Yes	226,995 (13.4)	4537 (14.7)	1.1 (1.1-1.2)	1.1 (1.0-1.1)
No	1,466,987 (86.6)	26,380 (85.3)	1.0 (ref.)	1.0 (ref.)
**Pre-pregnancy diabetes**				
Yes	39,415 (2.3)	886 (2.9)	1.2 (1.1-1.3)	1.1 (1.0-1.1)
No	1,654,567 (97.7)	30,031 (97.1)	1.0 (ref.)	1.0 (ref.)
**Chronic hypertension**				
Yes	47,507 (2.8)	1253 (4.1)	1.4 (1.3-1.5)	1.2 (1.1-1.3)
No	1,646,475 (97.2)	29,664 (95.9)	1.0 (ref.)	1.0 (ref.)

[a]Except for the categorized maternal age at delivery, all other variables were adjusted for mode of conception, and maternal age at delivery. The categorized maternal age was only adjusted for mode of conception.

© 2024 Velez MP et al. *JAMA Network Open.*

eTable 4. Additional Analysis 2: Association Between Mode of Conception and Risk of Multifetal Pregnancy Reduction, as Well as Preterm Birth, in Ontario, Canada, From April 1, 2006, to March 31, 2021

Outcome	No. with outcome/ No. at risk	Rate (%, 95% CI)	Unadjusted relative risk (95% CI)	Adjusted relative risk (95% CI)[a]
**Multifetal pregnancy reduction**				
Unassisted conception	363/1,670,825	0.02 (0.02-0.02)	1.0 (ref.)	1.0 (ref.)
Ovulation induction or intrauterine insemination	176/24,395	0.7 (0.6-0.8)	33.1 (27.6-39.6)	26.7 (21.9-32.6)
In vitro fertilization	204/29,679	0.7 (0.6-0.8)	31.5 (26.5-37.4)	20.7 (16.6-25.8)
**Preterm birth < 37 weeks' gestation**				
Unassisted conception	117,505/1,670,825	7.0 (7.0-7.1)	1.0 (ref.)	1.0 (ref.)
Ovulation induction or intrauterine insemination	3264/24,395	13.4 (13.0-13.8)	1.9 (1.8-2.0)	1.7 (1.7-1.8)
In vitro fertilization	5047/29,679	17.0 (16.6-17.4)	2.4 (2.3-2.5)	2.2 (2.1-2.3)
**Preterm birth < 34 weeks' gestation**				
Unassisted conception	29,410/1,670,825	1.8 (1.7-1.8)	1.0 (ref.)	1.0 (ref.)
Ovulation induction or intrauterine insemination	1004/24,395	4.1 (3.9-4.4)	2.3 (2.2-2.5)	2.1 (1.9-2.2)
In vitro fertilization	1610/29,679	5.4 (5.2-5.7)	3.1 (2.9-3.2)	2.6 (2.5-2.8)

[a]Adjusted for maternal age at delivery, income quintile, immigration status, obesity, parity, pre-pregnancy diabetes and chronic hypertension.

© 2024 Velez MP et al. *JAMA Network Open.*

eFigure. Study Flowchart

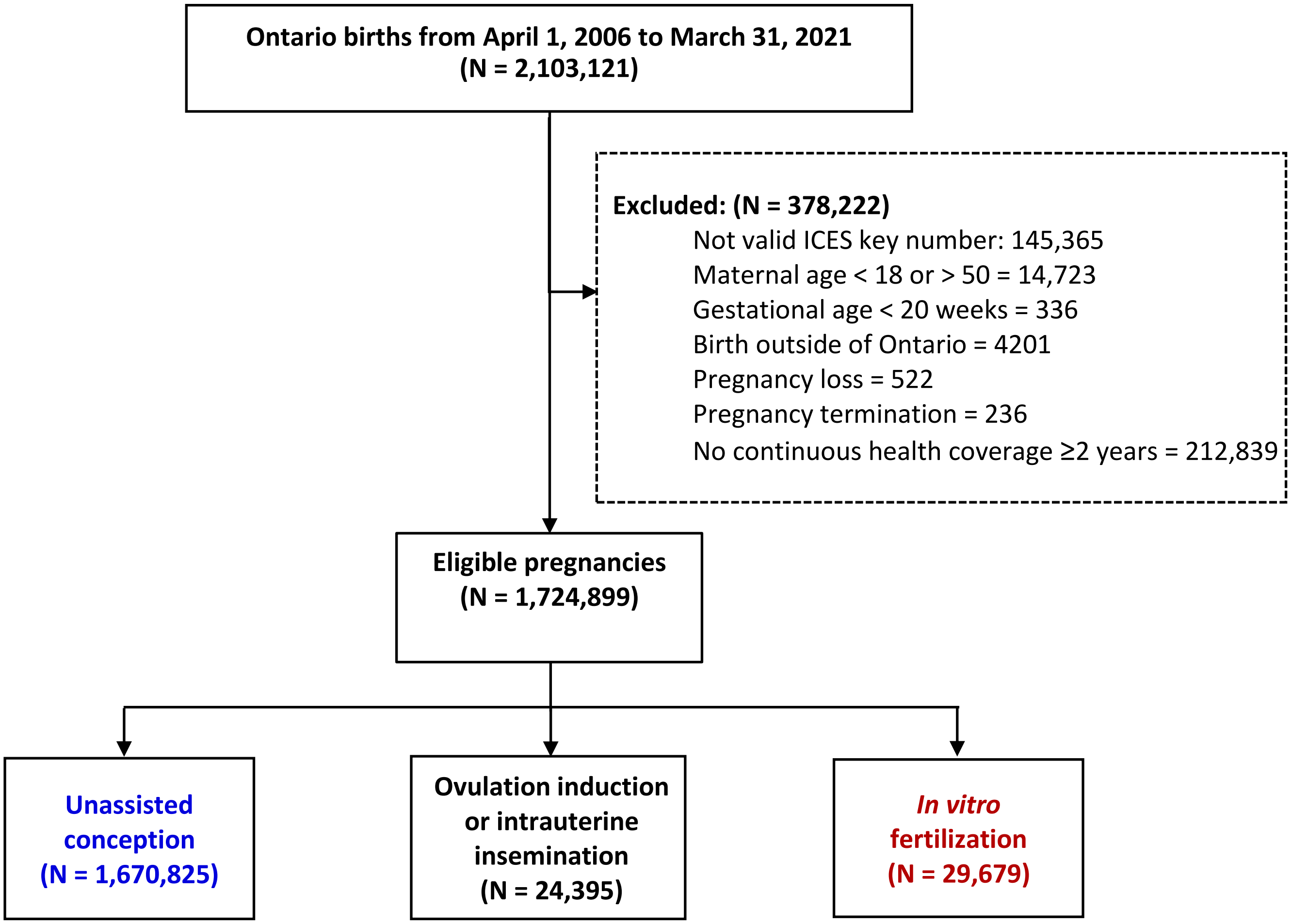

© 2024 Velez MP et al. *JAMA Network Open.*
