# Supplementary material for: Multifetal Pregnancy After Implementation of a Publicly Funded Fertility Program
Source: JAMA Netw Open. 2024 Apr 25;7(4):e248496. doi: 10.1001/jamanetworkopen.2024.8496 (PMC11046352; doi:10.1001/jamanetworkopen.2024.8496)
Supplement: Supplement 2. — Data Sharing Statement [file jamanetwopen-e248496-s002.pdf]

Data Sharing Statement

Velez. Fertility Treatment and Multifetal Pregnancy. *JAMA Netw Open*. Published April 25, 2024. doi:10.1001/jamanetworkopen.2024.8496

Data
Data available: No

Additional Information
Explanation for why data not available: The data set from this study is held securely in coded form at ICES. While data-sharing agreements prohibit ICES from making the data set publicly available, access may be granted to those who meet prespecified criteria for confidential access, available at www.ices.on.ca/DAS. The full data set creation plan and underlying analytic code are available from the authors upon request, understanding that the computer programmes may rely upon coding templates or macros that are unique to ICES and therefore either inaccessible or requiring modification.
